# Erroneous Belief that Digestive Stability Predicts Allergenicity May Lead to Greater Risk for Novel Food Proteins

**DOI:** 10.3389/fbioe.2021.747490

**Published:** 2021-09-17

**Authors:** Rod A. Herman, Jason M. Roper

**Affiliations:** ^1^ Corteva Agriscience, Indianapolis, IN, United States; ^2^ Corteva Agriscience, Newark, DE, United States

**Keywords:** allergens, digestion, risk, sensitization, tolerization, empirical evidence, mechanisms, exposure

## Abstract

There continues to be an erroneous belief that allergens (especially food allergens) are more resistant to gastrointestinal digestion than non-allergens. Government regulations based on this erroneous belief may result in technology developers altering the amino acid sequences of digestively stable native proteins to create digestively unstable modified versions for expression in genetically engineered crops. However, an investigation where a known stable allergen was modified to make it more digestible eliminated the protein’s ability to tolerize against allergy in a mouse model, which is consistent with the dual allergen exposure hypothesis. Thus, the false belief that digestive stability increases the allergenic risk of novel food proteins (e.g., such as expressed in genetically engineered crops) could, in some cases, lead to introduction of digestively unstable modified protein versions with greater sensitization risk. However, it is noteworthy that developers have historically been very effective at preventing allergens from being introduced into crops based on the other components of the weight-of-evidence assessment of allergenic risk such that no newly expressed protein in any commercialized genetically engineered crop has ever been documented to cause allergy in anyone.

## Introduction

Historically, it was thought that food allergens primarily sensitize individuals due to gut exposure followed by elicitation of food allergy after subsequent consumption of the food containing the offending protein (Kimber and Dearman, 2002). This led to the hypothesis that food allergens would be more stable to gastro-intestinal digestion compared with non-allergens. In 1996, a paper comparing the *in vitro* gastric digestibility of a group of allergenic and non-allergenic proteins was published that seemingly confirmed this expectation (Astwood et al., 1996). The observation that those taking acid-suppressant medications exhibited higher rates of allergy also appeared consistent with reduced gastric digestion (due to raised gastric pH slowing the action of pepsin in the stomach) increasing allergenic risk (Untersmayr and Jensen-Jarolim, 2008; Pali-Schöll and Jensen-Jarolim, 2011). Together this evidence reinforced the intuitive appeal that reduced digestion in the gut should increase the risk that a novel food protein would become an allergen (Herman et al., 2020).

### Empirical Evidence Showing That Allergens are not More Stable Than Non-allergens

As other researchers attempted to expand on the early digestive evaluation using greater numbers of allergens and non-allergens, it became clear that the purported correlation between digestive stability and allergenicity did not hold (Fu et al., 2002; Herman et al., 2007). It appeared that the selection of allergens and non-allergens evaluated by Astwood and colleagues (1996) confounded the protein function with the allergenicity status of the proteins (Schnell and Herman, 2009). When additional proteins were selected based on considerations for protein function and family, no discernable correlation between digestive stability and the allergenic status of proteins remained (Akkerdaas et al., 2018). It has also been recently observed that oral intake of known allergens at an early age reduces allergy later in life confirming that exposure in the gut is important to tolerization against allergy (Logan et al., 2020).

### Mechanistic Reasons why Increased Gut Exposure Does not Necessarily Favor Sensitization

The developing evidence that sensitization to food allergens can occur through dermal and inhalation exposure to food dust helps explain why gastro-intestinal digestive stability does not predict allergenic risk (Herman and Ladics, 2018). It is now hypothesized that initial sensitization to food allergens may occur *via* inhalation or dermal exposure to food dust (or through food handling or application of cosmetics containing crop by-products), followed by elicitation of allergy due to subsequent consumption of the food, and that initial gut exposure to the relevant protein in food may favor tolerization against allergy (dual allergen exposure hypothesis) (Kulis et al., 2021; Yakaboski et al., 2021).

The modern understanding that the state of human microbiome has a great impact on allergy development also helps explain why acid-suppressant medications increase allergy rates (Robinson and Camargo, 2018). It is now understood that the effect of acid-suppressant medications on the microbiome not only results in an increase in the frequency of food allergy, but also dermal and inhalation allergy (Robinson and Camargo, 2018). Clearly, the digestive fate of allergens in the gut is not relevant to eczema or rhinitis, and thus decreased digestion due to intake of acid-suppressant medications cannot explain why the frequency of allergy increases *via* all three exposure pathways. Similar to antibiotics, alteration of the human microbiome is the likely mechanism by which acid-suppressant medications increase allergy rates (Jordakieva et al., 2019; Herman, 2020; Herman et al., 2020).

### Reducing Exposure in the Gut can Eliminate Tolerization Against Allergy

An example has recently been published where modifying an allergenic protein to decrease its digestive stability might actually increase its allergenic risk. In this study, the digestive stability of a known food allergen (carp parvalbumin Cyp c 1) was reduced by modifying its amino acid sequence (Freidl et al., 2020). In a mouse model, it was found that oral exposure to the digestively stable native allergen tolerized mice against allergy, while the version modified to be less digestively stable did not. Mice initially exposed to the stable native allergen did not exhibit allergenic symptoms when later exposed to the proteins in food, while those mice exposed to the version modified to be more digestible were subsequently found to display allergenic symptoms. Thus, modifying a food protein with an allergenic risk to be less digestively stable could increase sensitization to that protein (Herman et al., 2021).

### Engineering Novel Food Proteins to be More Digestible Could Increase Allergenic Risk

**FIGURE 1:**
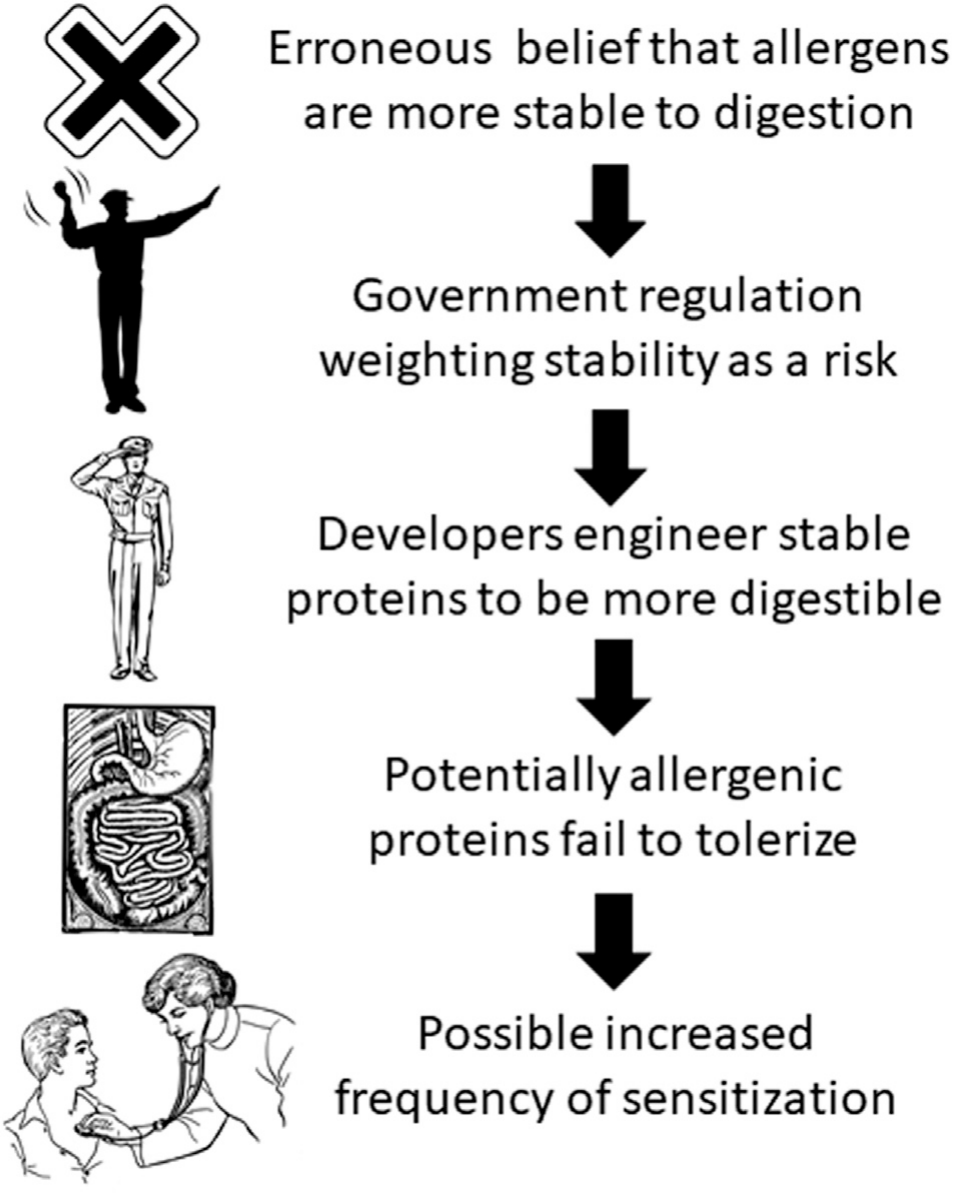
Possible result of erroneous belief that allergens are more stable to digestion than non-allergens (artwork from https://publicdomainvectors.org/en/public-domain/).

The preceding example highlights the potential risk of government regulation for genetically engineered crops based on the erroneous belief that gastro-intestinal digestibility decreases allergenic risk. Current government regulations for newly expressed proteins in genetically engineered crops universally consider gastro-intestinal stability to be a risk factor for allergenicity (Ladics, 2008). Thus, technology developers that discover proteins that would be useful if expressed in genetically engineered crops determine the digestive stability of these proteins in standardized *in vitro* digestion assays. If the protein is found to be stable in these assays, developers are motivated to modify the amino acid sequence to be more digestible while maintaining its beneficial activity (Parisi et al., 2020). While developers evaluate other components of the weight-of-evidence allergenicity assessment (e.g., bioinformatics, history of safe exposure, concentration in food, etc.) to minimize allergenic risk, the erroneous belief that digestibility is associated with reduced allergenic risk could, in some cases, increase the allergenic risk by reducing the tolerizing properties of a protein in the gut (Figure 1).

## Discussion

The current scientific evidence does not indicate a positive or negative correlation between gastro-intestinal digestion and the allergenic status of proteins (Bøgh and Madsen, 2016; Verhoeckx et al., 2019; Herman et al., 2020). Government regulations for newly expressed proteins in genetically engineered crops should take into account this scientific understanding. Here we have highlighted why outdated regulations that consider digestive stability a risk factor for allergenicity could create increased allergenic risk in some cases, and thus regulations should be updated to reflect the current preponderance of scientific evidence. However, it is noteworthy that developers have historically been very effective at preventing allergens from being introduced into crops based on the other components of the weight-of-evidence assessment of allergenic risk such that no newly expressed protein in any commercialized genetically engineered crop has ever been documented to cause allergy in anyone (Dunn et al., 2017). Finally, it is additionally noteworthy that digestion can decrease exposure in the gut and reduce symptoms in those already sensitized to an allergen, but bioinformatic comparisons between newly expressed protein amino-acid sequences and known allergens are conducted to determine cross-reactive risk, and if such a risk is indicated, serum screening is conducted to determine if cross reactivity is present. The results of the serum screening will determine if an elicitation risk is present irrespective of the digestibility of the new food protein. If a positive serum screening result occurs, the protein will not be developed even if it is readily digestible. Thus, there is no reasonable scenario where digestion results will determine the acceptability of risk.

## Data Availability

The original contributions presented in the study are included in the article, further inquiries can be directed to the corresponding author.
